# A Face-to-Face Surgical Instrumentation Course During the COVID-19 Pandemic

**DOI:** 10.7759/cureus.19266

**Published:** 2021-11-04

**Authors:** Samuel E Cullen, Angela Tiu, Kalpesh R Vaghela, Alistair R Hunter

**Affiliations:** 1 Trauma and Orthopaedics, University College London Hospital, London, GBR; 2 Trauma and Orthopaedics, Royal London Hospital, London, GBR

**Keywords:** covid-19, surgical instrumentation, surgical education, covid-19 medical education, core surgical training

## Abstract

Objectives

Surgical instrumentation teaching is included as an essential part of surgical training in the core surgical training syllabus. Access to formal teaching is variable, and opportunities for informal teaching have been further reduced by the COVID-19 pandemic. We aimed to design a course to fulfil these local trainees' needs. A move away from face-to-face teaching has occurred successfully during the pandemic, but little literature exists on how face-to-face courses can be best designed during this time. We aimed to describe the practicalities of running a face-to-face course with COVID restrictions.

Methods

Junior doctors and nurses rotated around five stations led by theatre nurses and senior doctors, each with common instruments from different surgical subspecialties. Social distancing was observed, and level 2 personal protective equipment (PPE) was worn throughout the course. Matched pre- and post-course tests allowed evaluation of learning.

Results

The course had 20 attendees, and the test scores improved following the course by an average of 9% (p = 0.009). All attendees (100%) found the course improved their knowledge and confidence. Feedback was overwhelmingly positive, and the significant improvement in the multiple-choice question (MCQ) scores demonstrates that this was an effective method of delivering teaching despite the COVID-19 restrictions on social distancing.

Conclusion

This course shows that instrumentation training is valuable to trainees and provides a good example to other educators, showing the workings of how a practical course may be run face-to-face during the pandemic.

## Introduction

Surgical instrumentation teaching is an essential part of a core surgical trainee’s education [1]. It is therefore important that junior surgical trainees across all disciplines are given access to formal teaching in a multi-disciplinary environment. This can be challenging due to the plethora of different instruments available and their often-eponymous names, which can be difficult to recall. At present, instrumentation teaching is not a part of surgical trainee inductions in all UK hospital Trusts.

A trainee’s experience of surgical instrumentation differs depending on the time spent in theatre and the availability and quality of teaching. Operating theatre exposure for junior trainees is increasingly limited; a recent large-scale audit demonstrated that only 5% of orthopaedic core surgical trainees met the Joint Committee on Surgical Training standards for minimum weekly clinical exposure [2]. The COVID-19 pandemic has had a detrimental impact on trainees’ surgical exposure, with elective surgery cancellations, limitations on theatre staff numbers and reduced training opportunities [3]. Some surgical centres reported consultant surgeons taking over simpler “training cases” to reduce operative times and transmission risks [4], which may reduce the time for intraoperative teaching. Formal instrumentation teaching will prepare trainees more effectively for theatre sessions and potentially equips trainees to use limited theatre time in a more focused and targeted manner.

Given the lack of specific surgical instrument teaching sessions at our university teaching hospital, a course was developed to introduce trainees to instruments they may commonly encounter. Two sessions were run for junior doctors (Foundation Year (FY) 1/2, Core Surgical Training Year 1) and junior theatre nursing staff. The course involved a structured teaching program with clear learning objectives.

COVID-19 has resulted in many successful adaptations to traditional teaching courses to minimise social contact and reduce viral transmission risk. Examples of this include online learning [5,6], remote/virtual simulation [3,7] and take-home learning kits for practical skills [8]. This course was delivered in September 2020, after the first peak of the COVID-19 pandemic in the United Kingdom, and there were benefits to running this course face-to-face, including live feedback and questioning during trainee instrument interaction and the ability to practice practical skills. This course provides an example of measures that can be used to ensure the safe delivery of face-to-face courses, which are compliant with social distancing guidelines.

The primary aim of this prospective study was to determine whether lecture- and practical-based instrument workshop teaching led to a significant improvement in knowledge. The secondary aim was to assimilate qualitative evidence about the strengths and weaknesses of the teaching methods used. We hypothesised that the course participants would achieve higher scores as a result of the teaching session.

## Materials and methods

Two surgical instrumentation teaching sessions were held in the hospital’s education centre in September 2020. The inclusion criteria were the completion of the pre- and post-teaching assessments, full attendance of the teaching session and being either a doctor at Foundation Year 1 or Senior House Officer grade or a junior nurse working in operating theatres. One attendee was excluded from the study, as both pre- and post-course feedback were not completed. Institutional board review was not possible at the time of the study due to the COVID-19 pandemic. Independent review from the Departmental Audit and Research Lead advised no ethical approval was required. Informed consent was gained from participants for the data to be published.

The education centre provided advice for courses carried out during the pandemic according to government social distancing guidelines. Firstly, course numbers were limited depending on room size to enable appropriate two-meter distancing according to UK government guidelines. The room used for the instrumentation course was risk assessed, allowing for a maximum of 18 people (13 attendees and five faculty). Secondly, level 2 personal protective equipment (PPE) was worn throughout the course, including face masks, aprons and gloves. The course was completed pre-rollout of the COVID-19 vaccine.

The attendees were split into five groups and allocated to one of five demonstration tables. These stations were neurosurgery, head and neck (ear, nose and throat; maxillofacial), orthopaedics, general surgery and suturing. Each station consisted of demonstration and practical hands-on style teaching, so attendees could learn the names and uses for commonly used instruments within each specialty. The surgical models used in some stations allowed attendees to practice psychomotor skills such as knee arthroscopy and perform an approach to a craniotomy using the instruments they had been shown. The teaching was led by senior theatre nurses or surgeons. All stations were run for 20 minutes before alcohol gel was distributed and attendees rotated to the next station.

An online 15-minute knowledge-based pre-course questionnaire was completed prior to starting the teaching stations. Following the completion of all five stations, attendees completed an online post-course questionnaire. This included Kirkpatrick level 1 subjective questions designed to gather information on how satisfied the attendees were with the way the course had been delivered. They then repeated the 15-minute questionnaire taken pre-course.

Kirkpatrick identified six levels of medical and surgical education assessment. We used online pre- and post-course questionnaires (SurveyMonkey Inc.) that combined two Kirkpatrick levels to assess the effectiveness of our course. The questionnaires collected non-specific personal data to enable anonymous pre- and post-course feedback matching.

Kirkpatrick level 1 analysis

The post-course questionnaire distributed to attendees collected subjective qualitative feedback. The response options were strongly agree, somewhat agree, neither agree nor disagree, somewhat disagree and strongly disagree.

Kirkpatrick level 2b analysis

The same 26-part multiple-choice questions (MCQs) were completed by the attendees as part of both pre- and post-course questionnaires. There were five questions each on head and neck, and neurosurgery instruments and four questions each on orthopaedics and general surgery instruments, which were being demonstrated during the course. The last eight questions were images that the attendees had to correctly name from a list of options.

The mean pre- and post-course scores were calculated and compared. The attendees’ pre- and post-course scores were analysed using a paired Student’s t-test (www.statskingdom.com). A p value < 0.05 was deemed to be a statistically significant difference in pre- and post-course scores.

## Results

The 20 attendees consisted of six foundation year doctors, 10 junior middle-grade doctors (core surgical trainees or surgical junior clinical fellows) and four junior theatre nurses (Table 1).

**Table 1:** Roles of the Attendees of the Surgical Instrument Teaching Day 2020

Role of Attendees	Number of Course Participants
Foundation year doctor (FY 1/2)	6
Junior middle-grade doctor (core surgical trainee/junior clinical fellow)	10
Theatre nurse	4

**Figure 1:**
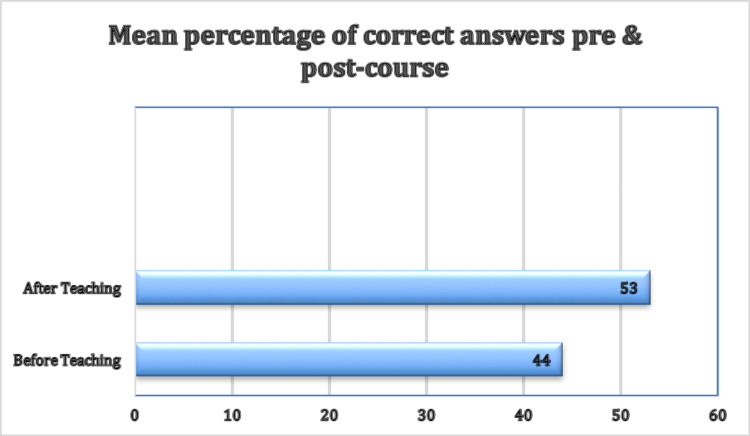
Mean Percentage of the Multiple-Choice Question Test Results Pre- and Post-Teaching During the Surgical Instrument Teaching Day 2020

Pre- and post-course MCQs were marked. The mean pre-course MCQ score was 44%, with post-course scores showing an improvement of up to 53% (Figure 1). A paired t-test demonstrated that this was a significant improvement following the course (p = 0.009).

**Figure 2:**
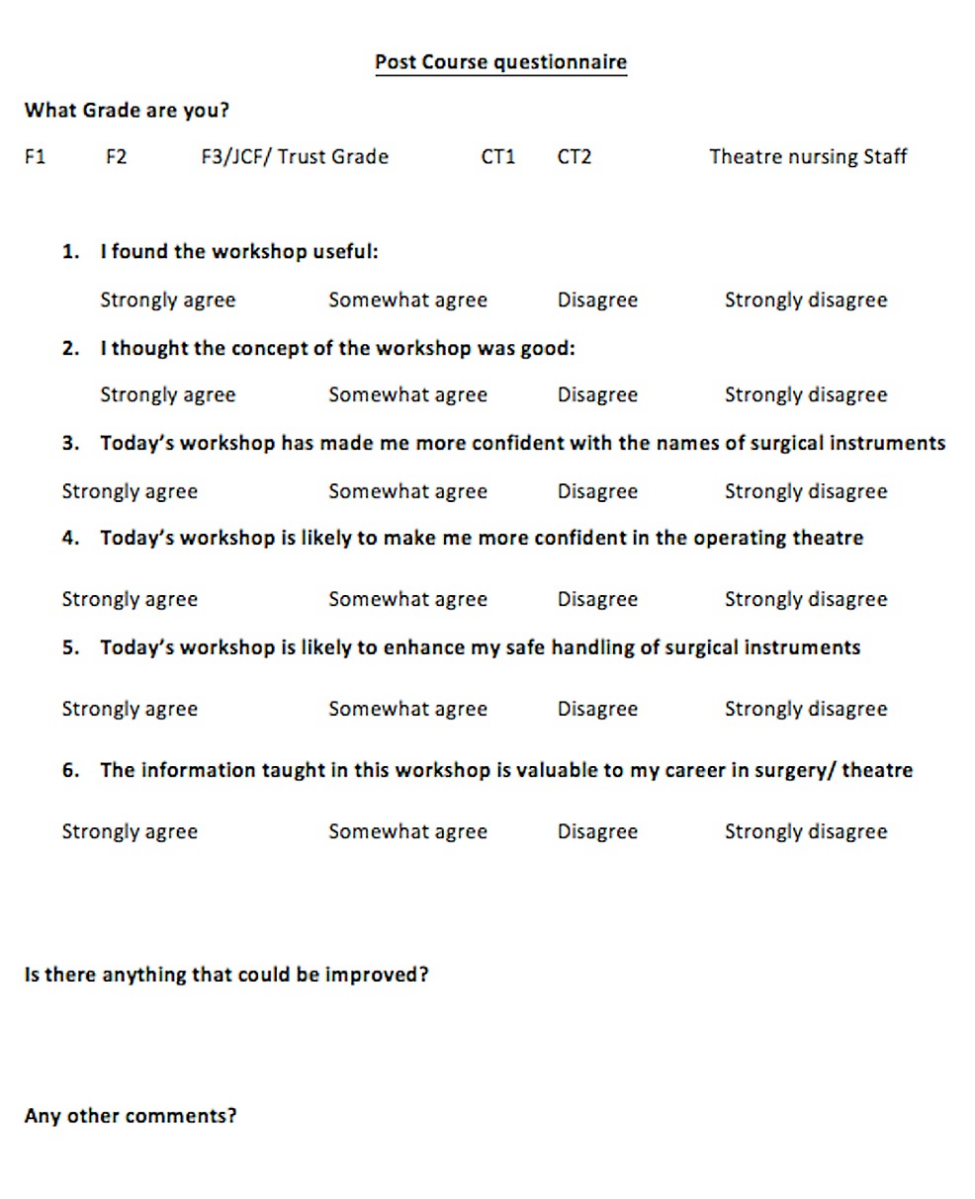
Qualitative Feedback Questions

**Figure 3:**
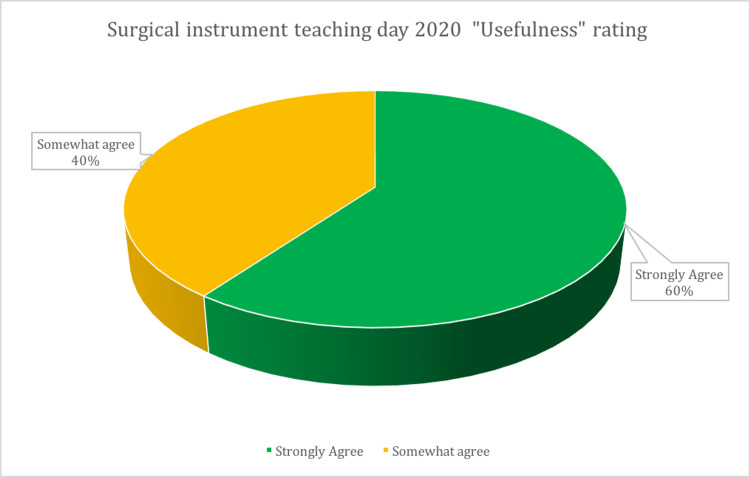
“Usefulness” Rating of the Surgical Instrument Teaching Day 2020 Reported by 20 Attendees

Attendee feedback was sought via qualitative feedback questions (Figure 2). Feedback was overwhelmingly positive, with 100% of attendees strongly (60%) or somewhat (40%) agreeing that they found the course useful (Figure 3) and improved their confidence and handling of surgical instruments. Of the attendees, 95% reported that they thought the course was valuable for their future careers (Table 2).

**Table 2:** Feedback From Attendees of the Surgical Instrument Teaching Day 2020

Attendee Feedback Statements	Number of Attendees in Agreement (/Total Attendees)
I found the workshop useful.	20/20
I thought the concept of the workshop was good.	20/20
Today’s workshop had made me more confident with the names of surgical instruments.	20/20
Today’s workshop is likely to make me more confident in the operating theatre.	20/20
Today’s workshop is likely to enhance my safe handling of surgical instruments.	20/20
The information taught in this workshop is valuable to my career in surgery/theatre.	19/20

Feedback was sought from the attendees on the aspects of the course that could be improved. The most common suggestion, which was made by 35% of the attendees, was the provision of handouts, with 25% of the attendees asking for more time at each station.

## Discussion

Introducing doctors in training and theatre nursing staff to the theatre setting involves explaining key instrumentation that they would be expected to use intraoperatively. Teaching is typically delivered in a lecture-based setting with little opportunity for participants to ask questions or appreciate practical elements. Optimising this training is vital to ensure that trainees feel supported and well prepared for their role.

Instrumentation training is on the syllabus for core surgical training but is not taught in many training centres. The results of this course show that the trainees did not only find it useful and valuable to their future careers but also showed a demonstrable improvement in their knowledge following their attendance. This provides evidence that instrumentation courses are well received by trainees, improve knowledge and provide formal education, meeting the core surgical training syllabus requirements.

Many teaching programs or courses have transitioned to online or remote methods during the COVID pandemic. Online practical skills courses have significant limitations; for example, it is hard to replicate the psychomotor skills taught on face-to-face courses remotely. A systematic review of the challenges and innovations during the pandemic outlines many different methodologies for transitioning training away from higher-risk face-to-face environments [3]. A hybrid approach may be an optimal solution for practical skills courses where the elements of the course can be taught remotely. The time spent in close quarters could be minimised when remote learning methods, such as online learning, remote simulation and take-home practical kits, are used and when a face-to-face practical element is combined, thus reducing transmission risk. Despite this, there were no aspects of this course that we felt would be effectively delivered remotely. The instrument demonstration benefitted from attendee interaction with the instruments and a chance to use them during practical aspects of the stations under supervision. The measures used during this course therefore present an example of how a practical skills course can be run during the pandemic whilst adhering to social distancing guidelines.

In this course, theatre nursing staff were responsible for the majority of the teaching. Multidisciplinary teaching in this course was an opportunity to harness the extensive clinical and technical experience of surgical instrumentation. Feedback was overwhelmingly positive, and the significant improvement in MCQ scores demonstrates that this was an effective method of delivering teaching.

A limitation of this study was its small sample size (20 participants). The number of delegates was limited by the COVID-19 social distancing regulations. A larger number would yield increased feedback and data to analyse learning response to teaching and a wealth of evidence on which to base future changes to the course.

## Conclusions

This course combined practical demonstration and lecture-based teaching at specialty-themed stations to deliver effective teaching for an essential part of the core surgical curriculum. The course was delivered in September 2020, after the first peak of the COVID-19 pandemic in the United Kingdom, and there were benefits to running this course face-to-face, which included live feedback and questioning during trainee instrument interaction and the ability to practice practical skills. Significant efforts were made to accommodate the COVID-19 social distancing restrictions. The results of the pre- and post-course scores demonstrate a statistically significant improvement in knowledge. The results are relevant because junior trainees require good instrumentation training to facilitate improved knowledge of and performance in the theatre environment, which are difficult to replicate in online teaching. This course therefore serves as an example of an effective method for teaching instrumentation, which was well received by trainees.

Our practical recommendations for running a course with social distancing restrictions are appropriate room size selection to allow for two-meter social distancing, an appropriate number of attendees to ensure no overcrowding, and wearing of level 2 PPE. Pre-course lateral flow tests could also act as an additional safeguard. The practical recommendations made in this study are likely to remain relevant as advice on social distancing and the restrictions on conducting face-to-face courses may continue. We therefore provide a practical method of continuing the delivery of such courses in this context.
